# Experimental Examination of Conventional, Semi-Automatic, and Automatic Volumetry Tools for Segmentation of Pulmonary Nodules in a Phantom Study

**DOI:** 10.3390/diagnostics14010028

**Published:** 2023-12-22

**Authors:** Julian Hlouschek, Britta König, Denise Bos, Alina Santiago, Sebastian Zensen, Johannes Haubold, Christoph Pöttgen, Andreas Herz, Marcel Opitz, Axel Wetter, Maja Guberina, Martin Stuschke, Waldemar Zylka, Hilmar Kühl, Nika Guberina

**Affiliations:** 1 Department of Radiotherapy, West German Cancer Center, University Hospital Essen, Hufelandstrasse 55, 45147 Essen, Germany; 2 Department of Radiology, University Hospital Muenster (UKM), Albert-Schweitzer-Campus 1, Gebäude A1, 48149 Muenster, Germany; 3 Institute of Diagnostic and Interventional Radiology and Neuroradiology, University Hospital Essen, Hufelandstrasse 55, 45147 Essen, Germany; 4 Department of Diagnostic and Interventional Radiology, Neuroradiology, Asklepios Klinikum Harburg, Eißendorfer Pferdeweg 52, 21075 Hamburg, Germany; 5 Westphalian University, Campus Gelsenkirchen, Neidenburger Str. 43, 45897 Gelsenkirchen, Germany; 6 Department of Radiology, St. Bernhard-Hospital Kamp-Lintfort, Bürgermeister-Schmelzing-Str. 90, 47475 Kamp-Lintfort, Germany

**Keywords:** radiation oncology, radiotherapy, target volume, contouring, semi-automation, N1 LUNGMAN phantom, lung cancer, pulmonary nodules, deep learning algorithm, Syngo.via RT image suite

## Abstract

The aim of this study is to examine the precision of semi-automatic, conventional and automatic volumetry tools for pulmonary nodules in chest CT with phantom N1 LUNGMAN. The phantom is a life-size anatomical chest model with pulmonary nodules representing solid and subsolid metastases. Gross tumor volumes (GTV_i_s) were contoured using various approaches: manually (0); as a means of semi-automated, conventional contouring with (I) adaptive-brush function; (II) flood-fill function; and (III) image-thresholding function. Furthermore, a deep-learning algorithm for automatic contouring was applied (IV). An intermodality comparison of the above-mentioned strategies for contouring GTV_i_s was performed. For the mean GTV_ref_ (standard deviation (SD)), the interquartile range (IQR)) was 0.68 mL (0.33; 0.34–1.1). GTV segmentation was distributed as follows: (I) 0.61 mL (0.27; 0.36–0.92); (II) 0.41 mL (0.28; 0.23–0.63); (III) 0.65 mL (0.35; 0.32–0.90); and (IV) 0.61 mL (0.29; 0.33–0.95). GTV_ref_ was found to be significantly correlated with GTV_i_s (I) *p* < 0.001, r = 0.989 (III) *p* = 0.001, r = 0.916, and (IV) *p* < 0.001, r = 0.986, but not with (II) *p* = 0.091, r = 0.595. The Sørensen–Dice indices for the semi-automatic tools were 0.74 (I), 0.57 (II) and 0.71 (III). For the semi-automatic, conventional segmentation tools evaluated, the adaptive-brush function (I) performed closest to the reference standard (0). The automatic deep learning tool (IV) showed high performance for auto-segmentation and was close to the reference standard. For high precision radiation therapy, visual control, and, where necessary, manual correction, are mandatory for all evaluated tools.

## 1. Introduction

While automatic contouring of organs at risk (OAR) has been extensively examined and is increasingly established in radiation therapy departments [1], the automatic delineation of targets remains a significant challenge, even in the context of online onboard adaptive planning [2]. Accurate target definition requires expertise to convert recommendations and clinical information into a high precision treatment plan. The aim of facilitating the auto-contouring of targets nonetheless seems attractive for many reasons, and continues to be a subject of ongoing research [3,4,5]. The issue of microscopic extension of malignant tumors is a crucial and challenging one in target volume definition in radiation therapy. Necessary margins need to be constantly reevaluated, particularly when advancing the development of medical techniques. In radiation therapy, planning concepts have moved away from using point prescription towards marginal prescription methods [6].

The most evident development in treatment planning is probably stereotactic radiation therapy. According to the treatment protocols of previous clinical trials, and following the current recommendations in the ESTRO guidelines, a 0 mm CTV margin is accepted for stereotactic radiotherapy in early-stage cancer., A 5 mm to 8 mm margin for curative irradiation is necessary for locally advanced non-small cell lung cancer [7,8,9].

Lung lesions show a large variation in size, location, involvement of surrounding tissues and contours. There are previous reports on algorithms for the segmentation of a wide variety of lung lesions, ranging from the large tumor formations found in patients with advanced lung cancer to the small nodules detected by lung cancer screening programs [10].

Many models for nodule classification have been examined and trained [11,12,13,14,15,16,17]. With respect to automatic contouring, the best agreement was found for lungs [18]. When characterizing nodules, it is crucial to examine how detection and auto-contouring tools implemented in clinical treatment planning systems function.

Nevertheless, questions regarding accurate segmentation and final verification persist and may vary across different contouring tools and techniques. Phantom measurements with dedicated examination of radiation therapy contouring tools are lacking.

Thus, we took as a reference for geometrical measurement an anthropomorphic phantom with precisely known implanted lung lesions. We semi-automatically contoured the nodules using a clinically certified treatment planning system.

## 2. Materials and Methods

Semi-automatic, conventional, and automatic segmentation of pulmonary lung nodules in an anthropomorphic phantom were contrasted and compared with the manually contoured geometrical volume as a reference standard. Additionally, the volumes provided by the manufacturer were paralleled.

### 2.1. Antrophomorphic Phantom

This study was conducted using an anthropomorphic multipurpose phantom, the N1 LUNGMAN phantom PH-1 R16511 (Kyoto Kagaku Co., Ltd., Kyoto, Japan). The phantom represents a life-size anatomical chest model (size: approx. 43W × 20D × 46H cm, chest girth: 94 cm, weight: approx. 18 kg, water-equivalent diameter: approx. 23.5 cm). Its anatomical components were calibrated using Hounsfield values (HU) corresponding to real human tissue. The chest wall comprises synthetic bones based on measurements of clinical data. The internal phantom components are heart, trachea, pulmonary vessels, abdomen (diaphragm) block, representing the upper abdomen. Furthermore, the phantom contains 9 pulmonary nodules, which represent metastases. In total, 3 pulmonary nodules are subsolid, and 6 pulmonary nodules are solid. Of note, spherical nodules without spiculae were used in the present phantom study, as described earlier [19,20]. The phantom was scanned in a supine and arms-abducted position, taken head first into the CT scanner, with a dedicated chest CT protocol. The CT scan was acquired using a multi-slice CT scanner (Siemens Healthineers, Erlangen, Germany) with a Stellar Infinity detector. The scan field of the chest covers the whole thorax, from the upper aperture to the diaphragm. The field of view was set to 380 mm. Scan length was set to 62 slices with a slice thickness of 5 mm for chest scans.

### 2.2. Radiation Oncology Workflow

When contouring GTV_i_s, viz. the phantom pulmonary nodules, CT imaging data was imported to Aria Oncology Information System^®^ (Varian Medical Systems Inc., Palo Alto, Santa Clara, CA, USA), which is similar to a workflow in clinical radiation oncology. First, the phantom pulmonary nodules were lined manually. Two radiation oncologists approved the manually drawn contours (0). These served as a reference GTV for comparison with the semi-automatically contoured GTV_i_s. Next, GTV_i_s were contoured by means of three semi-automatic, conventional tools. First, nodules were segmented using the “adaptive brush” function in the lung window (I). For the “flood fill”-GTV_i_s, flood fill volume growing intensity (%) was applied as indicated in Table 1 in the Results Section (II). Another tool for semi-automated conventional contouring was applied with the image thresholding function within the depicted density thresholds (HU) (Table 1) (III). For comparison of the above-mentioned strategies and contouring tools, Sørensen–Dice indices were calculated as described elsewhere [21].

Additionally, automatic contouring implemented in 3D Pulmo of the Syngo.via RT Image Suite (Siemens Healthineers, Forchheim, Germany)) was applied (IV). The automatic contouring algorithm is based on a convolutional neural networks (CNN) architectural workflow. CNN is used for feature computation for each potential lesion. First, the input image patch is processed by batch normalization. Subsequently, three blocks of operations are computed. In each block, a convolution with stride 2 is used for down-sampling instead of max-pooling. Semantic features from image features are computed using two fully connected layers. A soft-max function, when applied to each potential lesion, assigns 2 values corresponding to the probability of the finding being a nodule or a false positive. Finally, a weighted sum of the scores from this phase and the results from the prior step are computed. Findings above a certain threshold score are labeled as pulmonary nodules.

The performance of the contouring tools is classified on a 4-point scale, ranging from 0–3, categorized as minor if modifications are required to a few CT slices (<10%), intermediate if many slices require modification, and major if many slices require larger edits or the structure needs a complete recontouring

For intermodality, comparison contouring time and geometrical concordance (volume variation, Dice Similarity Coefficient (DSC)) were evaluated.

### 2.3. Statistical Analysis

Descriptive analysis was performed to compare the evaluated contouring tools. Kolmogorov–Smirnov and Shapiro–Wilk tests were used to examine normal distribution. The Wilcoxon test was performed to determine the intermodality difference. A *p*-value lower than 0.05 was considered statistically significant. Statistics were performed using SPSS, version 29.0.1.0 (IBM Corp., Armonk, NY, USA). Graphs were created using Prism version 9 (Graph Pad Inc., San Diego, CA, USA).

## 3. Results

**Figure 1:**
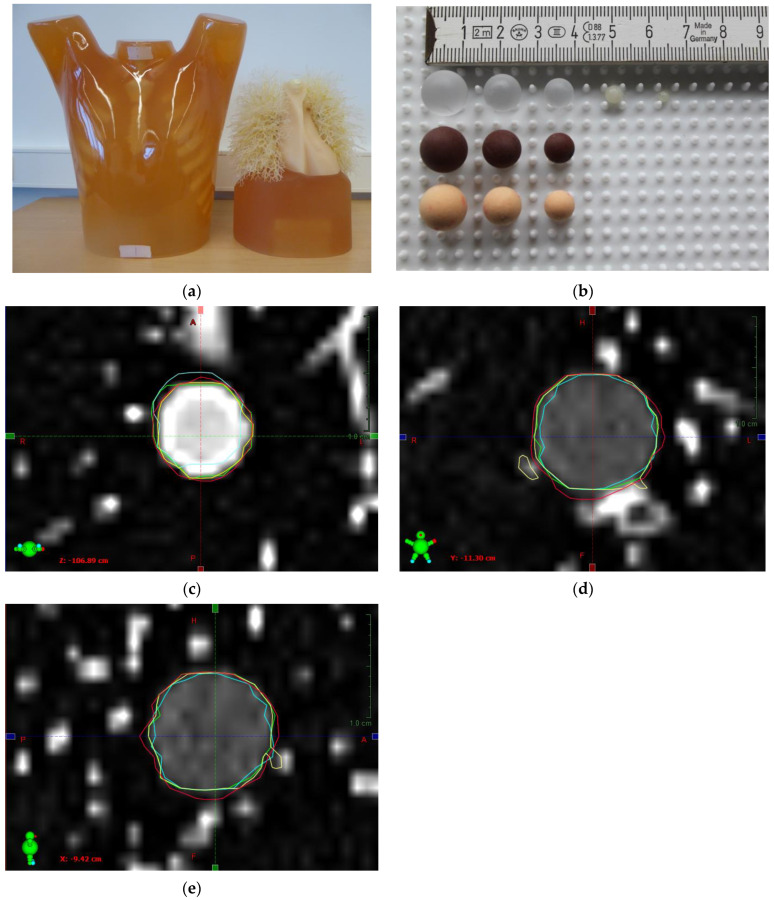
Representative images of the N1 LUNGMAN phantom (**a**,**b**) and CT scans of solid or subsolid lung nodules (**c**–**e**). (**a**) N1 LUNGMAN phantom overview. (**b**) Spherical nodules. (**c**) Solid nodule, transversal. (**d**) Subsolid nodule, frontal. (**e**) Subsolid nodule, sagittal. Different contouring techniques are indicated in red: manual contouring; green: adaptive brush function; cyan: flood fill function; yellow: image thresholding function. Scale bars: 1 cm.

For contouring of the pulmonary nodules (gross tumor volume (GTV_i_)), the CT imaging data was imported to Aria Oncology Information System^®^ (Varian Medical Systems Inc., Palo Alto, Santa Clara, CA, USA). GTV_i_s were contoured by various means: manually by two radiation oncologists (0), and as means of semi-automated, conventional contouring with (I) adaptive brush function, (II) flood fill function, and (III) image thresholding function. Furthermore, a deep-learning algorithm for automatic contouring was applied (IV). An intermodality comparison of the above-mentioned strategies for contouring GTV_i_s was performed with the manually contoured volume as the reference standard (GTV_ref_), and the manufacturer provided original volumes. Representative images of the N1 LUNGMAN phantom and CT images of solid and subsolid lung nodules are depicted in Figure 1.

**Figure 2:**
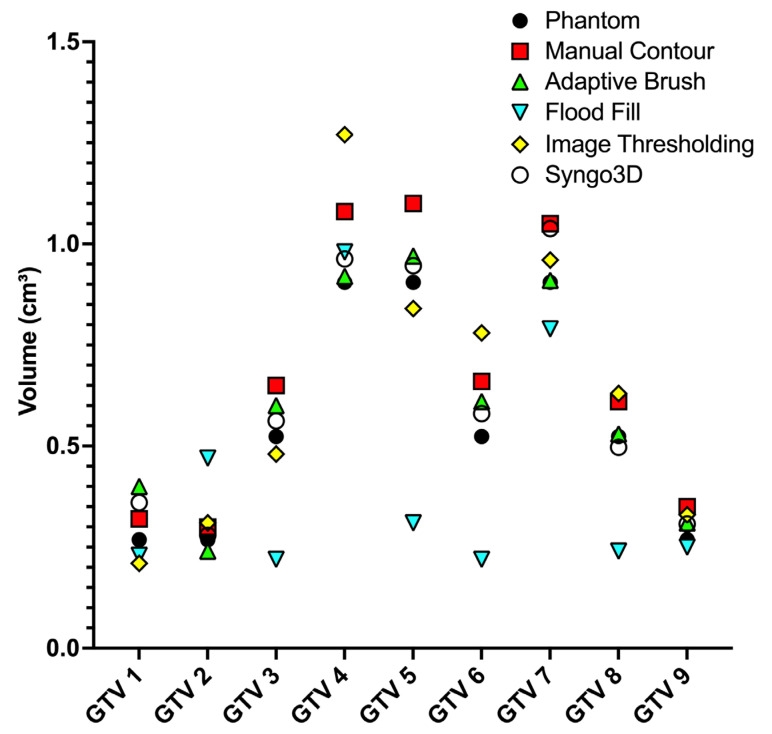
Individual volumes of the pulmonary nodules (GTV*_i_* 1–9) as described by the manufacturer (Phantom), the different contouring techniques (red: manual contouring; green: adaptive brush function; cyan: flood fill function; yellow: image thresholding function) and with a deep learning algorithm for automatic contouring (Syngo3D).

Mean GTV_ref_ (cm^3^) (standard deviation (SD); interquartile range (IQR)) was 0.68 (0.33; 0.34–1.1); GTV segmentation was distributed as follows: (I) 0.61 (0.27; 0.36–0.92); (II) 0.41 (0.28; 0.23–0.63); (III) 0.65 (0.35; 0.32–0.90); and (IV) 0.61 (0.29; 0.33–0.95) (Figure 2).

For the flood fill tool (II), a mean growing intensity of 21.7% (median 22.5%, range 15–27%) for segmentation of the solid nodules was applied. For (II) segmentation of the subsolid nodules, a mean growing intensity of 22.7% (median 26%, range 15–27%) was used. For the image thresholding tool (III) for segmentation of the solid nodules, a lower Hounsfield unit limit of mean −498.7 HU (median −478.0 HU, range −740.5–−298.4 HU) and an upper Hounsfield unit limit of mean 511.9 HU (median 537.3 HU, range 295.5–599.4 HU) was set. For (III) segmentation of the subsolid nodules, a lower Hounsfield unit limit of mean −827.9 HU (median −837.2 HU, range −892.4–−754.3 HU) and an upper Hounsfield unit limit of mean −244.4 HU (median −302.1 HU, range −312.3–−118.9 HU) was set (Table 1).

The intermodality comparison shows that GTV_ref_ correlates highly with the volume provided by the manufacturer, *p* < 0.001, r = 0.997 [95% CI: 0.988–0.999]. When differentiating the nodules according to texture, it emerged that the correlation of GTV_ref_ and the volume provided by the manufacturer were highly significant for both solid and subsolid volumes: *p* < 0.001, r = 0.999 and *p* = 0.009, r = 1.000. Detailed analysis revealed that overall GTV_ref_ significantly correlated with segmented GTV_i_s: (I) *p* < 0.001, r = 0.989 [95% CI: 0.946–0.998] and (III) *p* = 0.001, r = 0.916 ** [95% CI: 0.643–0.982], (IV) *p* = 0.001, r = 0.986, but not with (II) *p* = 0.091, r = 0.595 [95% CI: −0.114–0.903]. When differentiating the nodules according to texture, it emerged that the correlation of segmented solid GTV_i_s with GTV_ref_ was significant for (I) *p* < 0.001, r = 0.990, (III) *p* = 0.015, r = 0.897, (IV) *p* < 0.001, r = 0.994, but not with (II) *p* = 0.190, r = 0.619. Correlation of segmented subsolid GTV_i_s with GTV_ref_ was significant for (I) *p* = 0.014, r = 1.000, but not for (II) *p* = 0.543, r = 0.658 and (III) *p* = 0.058, r = 0.996, and (IV) *p* = 0.090, r = 0.990.

Overall, when differentiating different types of textures, viz. solid vs. subsolid, the exact Wilcoxon test showed that the volumes of solid nodules significantly differed between (II) and GTV_ref_, *p* = 0.031. There was no significant difference between the volumes of solid nodules created by (I), (III), (IV) and GTV_ref_, *p* = 0.219, *p* = 0.688, and *p* = 0.063. There was no significant difference between volumes of subsolid nodules created by (I), (II), (III) as well as (IV) and GTV_ref_, *p* = 0.250, *p* = 0.500, *p* = 1.000, and *p* = 0.250.

Sørensen–Dice indices were 0.74 overall for (I), 0.57 for (II), and 0.71 for (III) (Table 2). Differentiating solid and subsolid pulmonary nodules for solid GTVs Sørensen–Dice indices were 0.72 for (I), 0.55 for (II), and 0.70 for (III), and for subsolid GTVs Sørensen–Dice indices were 0.79 for (I), 0.62 for (II), and 0.74 for (III). Meanwhile, contour evaluation revealed that auto-segmented GTVs required either none or minor editing in 55.6% and 44.4% for (I); minor and major editing in 11.1% and 89.9% for (II); and intermediate and major editing in 77.8% and 22.2% for (III). Segmentation time was shortest for (I) compared to the other semi-automatic tools, *p* < 0.001. Segmentation time for (I) was 120 s, 180 s for (II), and 240 s for (III).

## 4. Discussion

The aim of this phantom study was to examine the precision of semi-automatic, conventional, and automatic volumetry tools for contouring pulmonary nodules with dedicated radiation treatment techniques in the chest for high-end multi-slice CT scans with the phantom N1 LUNGMAN. In the present study, of the semi-automatic conventional segmentation tools evaluated, the adaptive brush function performed closest to the reference standard. The evaluated automatic deep learning tool showed a high performance for auto-segmentation and was also close to the reference standard. Nonetheless, for the design of high precision radiation therapy treatment plans, a final visual control and potentially manual corrections remain mandatory for all evaluated tools. Currently, a particular challenge in the application of different segmentation tools is the need to specifically adjust the input parameters to obtain the desired results. Therefore, a thorough knowledge of anatomy and the workflow specifications is essential.

By default, the brush tool adapts to the grayscale values on the image plane and the brush diameter varies automatically in both 2D and 3D while drawing (ARIA OIS for RO version 16.0, Varian Medical Systems Inc., Palo Alto, CA, USA). The smallest brush diameter corresponds to the width of four image pixels for normal resolution structures or two image pixels for high-resolution structures. In contrast to the adaptive brush, the static brush does not adapt to the grayscale values on the image plane and the brush diameter does not change automatically while drawing. The adaptive brush tool performed best of all the semi-automatic segmentation tools examined, probably due to the option to immediately adjust the diameter automatically during the contouring procedure.

The flood fill tool is a conventional, semi-automatic contouring tool implemented in the treatment planning system that generates structures by merging adjacent pixels based on their similarity to an initial point. Connectivity is determined by the adjacent pixel, and a preset 2D or 3D volume growing intensity. The segmentation operation can be controlled by a growing factor, the effect of which can be visually verified (ARIA OIS for RO version 16.0, Varian Medical Systems Inc., Palo Alto, Santa Clara, CA, USA). Finding the proper volume growing intensity by systematic testing of different values (%) is a workflow challenge which makes the tool less applicable for the segmentation of particularly small lesions, such as pulmonary nodules. Likewise, this explains the longer time needed to complete the contouring process compared to the other tool evaluated.

Image thresholding is a segmentation tool designed for searching voxels with CT values within defined limits. The CT values in the image thresholding tool are shown by the units defined in the image (for example, HUs or pixels). It is important to visually identify the proper CT value range and then the tool automatically searches the voxels within that range. It partitions the input image by applying one or more cut-off values (thresholds) on the grey-level intensities (ARIA OIS for RO version 16.0, Varian Medical Systems Inc., Palo Alto, USA). A particular challenge for the imaging thresholding tool is to find the proper threshold for segmentation of pulmonary nodules. Furthermore, for different types of texture it is important to find the most appropriate grayscale range for accurate segmentation. As in the lung window, solid nodules are like the adjacent vessels, and they are often incidentally included in the gross target volume. Thus, manual post-processing correction of these contours is necessary. On the other hand, subsolid nodules and vessels are better discriminated by Hounsfield units. However, its margin to the surrounding parenchyma may be less clearly definable.

There are different automatic, computed aided techniques for the identification and classification of pulmonary nodules. Some help to detect pulmonary lesions, while others try to characterize the type of lesion. In the present study, the 3D Pulmo Syngo.via automatic segmentation tool was applied relying on deep learning. The automatic segmentation proved valid for pulmonary nodule segmentation. The lesion quantification tool implemented provides automatic 3D segmentation of lung nodules based on lung nodule texture. The workflow is designed as a computer-aided detection method and second reader toolkit to assist in the detection of pulmonary lesions during review of CT examinations of the chest (https://marketing.webassets.siemens-healthineers.com/1800000000080437/d0a5bae38837/syngo_lungcare-00080437_1800000000080437.pdf (accessed on 9 November 2023)).

Multiple studies have examined the performance of segmentation tools with different results, depending on the method and technique [1,11,12,13,14,15,17]. To segment different types of lung nodules correctly, a whole range of networks are put forward.

Pang et al. acknowledge that segmentation of tumors is far more challenging than segmentation of normal tissue [22]. The authors propose a unified and end-to-end adversarial learning framework for automatic segmentation of any kinds of tumors, including lung, liver, and kidney lesions identified from CT scans. These scans are called CTumorGAN, and consist of a Generator network and a Discriminator network. The authors state that their data may be generalized to address any kinds of tumor datasets with superior performance.

Zhang et al. propose an U-Net network, which has practical value in terms of helping radiologists segment lung nodules and diagnose lung cancer [23]. The authors claim that their proposed method represents the best segmentation performance in terms of Sørensen–Dice indices compared to previous studies, which assessed state-of-the-art techniques.

Kang et al. examined the classification of lung nodules using 3D multi-view convolutional neural networks with both chain architecture and directed acyclic graph architecture [11]. The authors conducted a classification according to benign, malignant and metastatic malignant nodules on CT images from Lung Image Database Consortium and Image Database Resource Initiative database (LIDC-IDRI). The authors concluded that the evaluated multi-view-one-network strategy may achieve a lower error rate than the one-view-one-network strategy.

Baldwin et al. validated a lung nodule convolutional neural network (LN-CNN) in 1187 patients with 5–15 mm nodules, achieving an AUC of 89.6% [15]. Likewise, Hunter et al. developed a radiomics signature to classify nodules according to malignancy risk [17].

Interestingly, Ardila et al. propose a deep learning-based algorithm to predict the risk of lung cancer in low-dose CT scans of the chest in patients undergoing screening examinations [13]. The authors state that their algorithm outperformed 6 radiologists included in a reader study, in terms of sensitivity and specificity, when prior CT imaging was not available in a relatively large number of cases (507).

In addition to lesion identification and classification, another important point is the maintenance of contouring quality. A large review on deep learning techniques, excluding segmentation and contouring tasks, showed a rather sober picture of artificial intelligence in clinical workflow [24]. According to Nagendran et al., the overall risk of bias was high in the majority of the evaluated studies, and adherence to reporting standards was suboptimal. Contrary to automatic segmentation tools, the semi-automatic, conventional tools examined in our study present radiation oncology contouring tools as part of a certified and clinically approved treatment planning system. They are used routinely in our clinic and they are established in clinical practice. Furthermore, the evaluated contouring tools, though semi-automatically applied, still do not comprise any deep learning, machine learning techniques or neuronal networks for contouring, but rather rely on the experience of a radiation oncologist.

From a clinical point of view, it is important to mention the limited data on the extension of microscopic lung tumor margins. In non-small-cell lung cancer, the potential expansion of CTV beyond radiographic visibility to include potential microscopic disease, and thus improve treatment outcomes, is under constant discussion [25,26]. The individual anatomical situation, for example, tumor formations adjacent to larger blood vessels, requires individual adaptation of contouring strategies [25]. Of note, there are uneven definitions of suitable CTV margins even in large-cohort clinical trials. For instance, in the RTOG 0813 study no expansion of the GTV for potential microscopic disease in early-stage lung cancer was used [27]. Additionally, a high inter-individual variability in contouring strategies was reported between clinicians [28]. In the present study, spherical pulmonary nodules without spiculae were used as comparable to lung metastases. In contrast to automated contouring tools for organs at risk [1], the use of auto-contouring tools for GTVs or CTVs is not yet a commonly established clinical routine.

Issues about semi-automatic and automatic segmentation are under constant debate, and performance may vary between manufactures and techniques. It must be assumed that a combination of different technical settings will lead to different results, while some settings are consistent between different CT scans. More robust retrospective and prospective studies will be required to ensure clinical applicability.

For this study, a thorax phantom was chosen to provide a test subject with stable size and composition for the evaluation of CT pulmonary nodules. The advantages of using this phantom are that it is similar to human patients, as its anatomical components are calibrated with the Hounsfield values of human tissue and last but not least, no real patient was exposed to ionizing radiation.

## 5. Conclusions

For high-precision radiotherapy, final validation of pulmonary nodule segmentation is essential for all tools evaluated. Of the semi-automatic tools evaluated, the adaptive brush function came closest to the reference standard. Likewise, the automatic deep learning tool showed a high performance in automatic segmentation and was close to the reference standard.

## Figures and Tables

**Table 1:** For the “flood fill”-GTV_i_s, flood fill volume growing intensity (%) was applied as indicated. Another tool for semi-automated, conventional contouring was applied with the image thresholding function within the depicted density thresholds (HU).

	Flood Fill Volume Growing Intensity (%)	Image Thresholding Lower Limit (HU)	Image Thresholding Upper Limit (HU)
GTV 1	20	−326.07	544.18
GTV 2	15	−754.29	−118.86
GTV 3	17	−298.44	295.54
GTV 4	26	−685.22	530.37
GTV 5	27	−312.25	599.44
GTV 6	25	−740.48	530.37
GTV 7	27	−837.17	−302.12
GTV 8	26	−892.43	−312.25
GTV 9	15	−629.97	571.81

**Table 2:** Calculated Sørensen–Dice indices for the contouring techniques. Manual contouring was considered the reference standard for calculation.

	Adaptive Brush	Flood Fill	Image Thresholding
GTV 1	0.39	0.58	0.60
GTV 2	0.74	0.60	0.62
GTV 3	0.85	0.46	0.74
GTV 4	0.79	0.80	0.75
GTV 5	0.81	0.41	0.73
GTV 6	0.80	0.43	0.65
GTV 7	0.83	0.78	0.82
GTV 8	0.79	0.47	0.77
GTV 9	0.70	0.63	0.71

## Data Availability

All data generated or analyzed during this study are included in this published article.

## References

[1] Chen W., Wang C., Zhan W., Jia Y., Ruan F., Qiu L., Yang S., Li Y. (2021). A comparative study of auto-contouring softwares in delineation of organs at risk in lung cancer and rectal cancer. Sci. Rep..

[2] Guberina M., Garcia A.S., Khouya A., Pöttgen C., Holubyev K., Ringbaek T.P., Lachmuth M., Alberti Y., Hoffmann C., Hlouschek J. (2023). Comparison of Online-Onboard Adaptive Intensity-Modulated Radiation Therapy or Volumetric-Modulated Arc Radiotherapy with Image-Guided Radiotherapy for Patients With Gynecologic Tumors in Dependence on Fractionation and the Planning Target Volume Margin. JAMA Netw. Open.

[3] Agazaryan N., Chow P., Lamb J., Cao M., Raldow A., Beron P., Hegde J., Steinberg M. (2020). The Timeliness Initiative: Continuous Process Improvement for Prompt Initiation of Radiation Therapy Treatment. Adv. Radiat. Oncol..

[4] Hernandez S., Nguyen C., Gay S., Duryea J., Howell R., Fuentes D., Parkes J., Burger H., Cardenas C., Paulino A.C. (2023). Resection cavity auto-contouring for patients with pediatric medulloblastoma using only CT information. J. Appl. Clin. Med. Phys..

[5] Zhong Y., Guo Y., Fang Y., Wu Z., Wang J., Hu W. (2023). Geometric and dosimetric evaluation of deep learning based auto-segmentation for clinical target volume on breast cancer. J. Appl. Clin. Med. Phys..

[6] Kawahara D., Saito A., Nagata Y. (2023). Physical and biological dosimetric margin according to prescription method for stereotactic body radiation therapy. J. Radiat. Res..

[7] Timmerman R., Paulus R., Galvin J., Michalski J., Straube W., Bradley J., Fakiris A., Bezjak A., Videtic G., Johnstone D. (2010). Stereotactic Body Radiation Therapy for Inoperable Early Stage Lung Cancer. JAMA.

[8] Timmerman R.D., Paulus R., Pass H.I., Gore E.M., Edelman M.J., Galvin J., Straube W.L., Nedzi L.A., McGarry R.C., Robinson C.G. (2018). Stereotactic Body Radiation Therapy for Operable Early-Stage Lung Cancer: Findings from the NRG Oncology RTOG 0618 Trial. JAMA Oncol..

[9] Nestle U., De Ruysscher D., Ricardi U., Geets X., Belderbos J., Pöttgen C., Dziadiuszko R., Peeters S., Lievens Y., Hurkmans C. (2018). ESTRO ACROP guidelines for target volume definition in the treatment of locally advanced non-small cell lung cancer. Radiother. Oncol..

[10] Tan Y., Schwartz L.H., Zhao B. (2013). Segmentation of lung lesions on CT scans using watershed, active contours, and Markov random field. Med. Phys..

[11] Kang G., Liu K., Hou B., Zhang N. (2017). 3D multi-view convolutional neural networks for lung nodule classification. PLoS ONE.

[12] Shaffie A., Soliman A., Fraiwan L., Ghazal M., Taher F., Dunlap N., Wang B., van Berkel V., Keynton R., Elmaghraby A. (2018). A Generalized Deep Learning-Based Diagnostic System for Early Diagnosis of Various Types of Pulmonary Nodules. Technol. Cancer Res. Treat..

[13] Ardila D., Kiraly A.P., Bharadwaj S., Choi B., Reicher J.J., Peng L., Tse D., Etemadi M., Ye W., Corrado G. (2019). End-to-end lung cancer screening with three-dimensional deep learning on low-dose chest computed tomography. Nat. Med..

[14] Massion P.P., Antic S., Ather S., Arteta C., Brabec J., Chen H., Declerck J., Dufek D., Hickes W., Kadir T. (2020). Assessing the Accuracy of a Deep Learning Method to Risk Stratify Indeterminate Pulmonary Nodules. Am. J. Respir. Crit. Care Med..

[15] Baldwin D.R., Gustafson J., Pickup L., Arteta C., Novotny P., Declerck J., Kadir T., Figueiras C., Sterba A., Exell A. (2020). External validation of a convolutional neural network artificial intelligence tool to predict malignancy in pulmonary nodules. Thorax.

[16] Hunter B., Hindocha S., Lee R.W. (2022). The Role of Artificial Intelligence in Early Cancer Diagnosis. Cancers.

[17] Hunter B., Chen M., Ratnakumar P., Alemu E., Logan A., Linton-Reid K., Tong D., Senthivel N., Bhamani A., Bloch S. (2022). A radiomics-based decision support tool improves lung cancer diagnosis in combination with the Herder score in large lung nodules. EBioMedicine.

[18] Radici L., Ferrario S., Borca V.C., Cante D., Paolini M., Piva C., Baratto L., Franco P., La Porta M.R. (2022). Implementation of a Commercial Deep Learning-Based Auto Segmentation Software in Radiotherapy: Evaluation of Effectiveness and Impact on Workflow. Life.

[19] König B., Guberina N., Kühl H., Zylka W. (2019). Design and first results of a phantom study on the suitability of iterative reconstruction for lung-cancer screening with low-dose computer tomography. Curr. Dir. Biomed. Eng..

[20] Bos D., König B., Blex S., Zensen S., Opitz M., Maier S., Forsting M., Zylka W., Kühl H., Wetter A. (2021). Experimental examination of radiation doses from cardiac and liver CT perfusion in a phantom study as a function of organ, age and sex. J. Radiol. Prot..

[21] Carass A., Roy S., Gherman A., Reinhold J.C., Jesson A., Arbel T., Maier O., Handels H., Ghafoorian M., Platel B. (2020). Evaluating White Matter Lesion Segmentations with Refined Sørensen-Dice Analysis. Sci. Rep..

[22] Pang S., Du A., Orgun M.A., Yu Z., Wang Y., Wang Y., Liu G. (2020). CTumorGAN: A unified framework for automatic computed tomography tumor segmentation. Eur. J. Nucl. Med. Mol. Imaging.

[23] Zhang X., Liu X., Zhang B., Zhang B., Dong J., Zhao S., Li S. (2021). Accurate segmentation for different types of lung nodules on CT images using improved U-Net convolutional network. Medicine.

[24] Nagendran M., Chen Y., Lovejoy C.A., Gordon A.C., Komorowski M., Harvey H., Topol E.J., Ioannidis J.P.A., Collins G.S., Maruthappu M. (2020). Artificial intelligence versus clinicians: Systematic review of design, reporting standards, and claims of deep learning studies. BMJ.

[25] Tamaki Y., Aibe N., Komiyama T., Nagasaka S., Imagumbai T., Itazawa T., Onishi H., Akimoto T., Nagata Y., Nakayama Y. (2022). Optimal Clinical Target Volume of Radiotherapy Based on Microscopic Extension around the Primary Gross Tumor in Non-Small-Cell Lung Cancer: A Systematic Review. Cancers.

[26] Grills I.S., Fitch D.L., Goldstein N.S., Yan D., Chmielewski G.W., Welsh R.J., Kestin L.L. (2007). Clinicopathologic Analysis of Microscopic Extension in Lung Adenocarcinoma: Defining Clinical Target Volume for Radiotherapy. Int. J. Radiat. Oncol. Biol. Phys..

[27] Bezjak A., Paulus R., Gaspar L.E., Timmerman R.D., Straube W.L., Ryan W.F., Garces Y.I., Pu A.T., Singh A.K., Videtic G.M. (2019). Safety and Efficacy of a Five-Fraction Stereotactic Body Radiotherapy Schedule for Centrally Located Non–Small-Cell Lung Cancer: NRG Oncology/RTOG 0813 Trial. J. Clin. Oncol..

[28] Spoelstra F.O., Senan S., Le Pechoux C., Ishikura S., Casas F., Ball D., Price A., De Ruysscher D., van Sornsen de Koste J.R. (2010). Variations in Target Volume Definition for Postoperative Radiotherapy in Stage III Non–Small-Cell Lung Cancer: Analysis of an International Contouring Study. Int. J. Radiat. Oncol..

